# Does Feedback-Related Brain Response during Reinforcement Learning Predict Socio-motivational (In-)dependence in Adolescence?

**DOI:** 10.3389/fpsyg.2016.00655

**Published:** 2016-05-06

**Authors:** Diana Raufelder, Rebecca Boehme, Lydia Romund, Sabrina Golde, Robert C. Lorenz, Tobias Gleich, Anne Beck

**Affiliations:** ^1^Ernst-Moritz Arndt University GreifswaldGreifswald, Germany; ^2^Charité – Universitätsmedizin BerlinBerlin, Germany; ^3^Max Planck Institute for Human DevelopmentBerlin, Germany

**Keywords:** fMRI, reinforcement learning, motivation, social relationships in school

## Abstract

This multi-methodological study applied functional magnetic resonance imaging to investigate neural activation in a group of adolescent students (*N* = 88) during a probabilistic reinforcement learning task. We related patterns of emerging brain activity and individual learning rates to socio-motivational (in-)dependence manifested in four different motivation types (MTs): (1) peer-dependent MT, (2) teacher-dependent MT, (3) peer-and-teacher-dependent MT, (4) peer-and-teacher-independent MT. A multinomial regression analysis revealed that the individual learning rate predicts students’ membership to the independent MT, or the peer-and-teacher-dependent MT. Additionally, the striatum, a brain region associated with behavioral adaptation and flexibility, showed increased learning-related activation in students with motivational independence. Moreover, the prefrontal cortex, which is involved in behavioral control, was more active in students of the peer-and-teacher-dependent MT. Overall, this study offers new insights into the interplay of motivation and learning with (1) a focus on inter-individual differences in the role of peers and teachers as source of students’ individual motivation and (2) its potential neurobiological basis.

## Introduction

### Inter-individual Differences in Students’ Scholastic Motivation

Studies in the field of educational psychology focus on the social school environment, which is mainly determined through relations with peers and teachers providing essential motivation (Harter, 1996). Indeed, several studies have shown that peers and teachers can play an important role for students’ scholastic motivation (Wentzel, 2009a,b, 2010), both individually and through a whole classroom approach (Pianta et al., 2003). This is particularly interesting during adolescence, when most students’ scholastic motivation declines (Harter, 1996; Zusho and Pintrich, 2001; Watt, 2004) due to changes in their environment or within themselves (Eccles et al., 1998; Wigfield and Eccles, 2001). Nevertheless, Wigfield and Eccles (2001) contend that some students do not necessarily reduce motivation (Deci and Ryan, 2002) suggesting that there are inter-individual differences in students’ motivation patterns based on environmental as well as developmental aspects. Previous person-oriented research (Raufelder et al., 2013c) investigated inter-individual differences in adolescents’ perception of peers and teachers as environmental sources of motivation, and proposed four distinct motivation types (MTs), which we discuss in more detail below.

### Four Different Motivation Types (MTs) and the Concept of Socio-motivational (In-)dependence

Raufelder et al. (2013b) differentiated four different MTs based on the importance of peers and teachers as sources for scholastic motivation. Applying a latent class analysis (LCA) in 1088 adolescent students from Germany they identified (1) teacher-dependent MT, (2) peer-dependent MT, (3) peer-and-teacher-dependent MT and (4) peer-and-teacher-independent MT (Raufelder et al., 2013b). Students of the teacher-dependent MT (1) receive most of their scholastic motivation from teachers. Further qualitative interviews (Hoferichter and Raufelder, 2014) revealed that these students are also affected by teachers’ own motivation as well as the support and feedback they perceive from the teacher. Likewise, students of the peer-dependent MT (2) are mostly driven by their peers, whereas students of the peer-and-teacher-dependent MT (3) perceive both peers and teachers as sources of motivation (Hoferichter and Raufelder, 2014). Finally, the motivation of students in the peer-and-teacher-independent MT category (4) remains largely unaffected by the motivation, learning behavior, or perceived support of their peers and teachers. The four MT have been validated by another LCA in a sample of adolescent students in Montréal, Canada (Hoferichter et al., 2014). Based on these findings Raufelder (2014) formulated the concept of socio-motivational (in-)dependence: individuals whose motivation is affected by others’ motivation, learning behavior or perceived support, are considered to be socio-motivationally dependent. In the school context, motivation can be affected by both peers’ motivation, learning behavior or social support and/or by teachers’ perceived support and motivation (see Wentzel, 2009a,b; Raufelder et al., 2015). In turn, when the motivation remains largely unaffected by others, socio-motivational independency is assumed. A subsequent longitudinal latent transition analysis (LTA) could confirm the four types of socio-motivational (in-)dependence by investigating intra-individual changes from early to middle adolescence (Jagenow et al., 2015). While slight turnovers were observed between the three types of socio-motivational dependence from early to middle adolescence, the socio-motivationally independent category showed the highest probability (0.68) to remain stable.

### Reinforcement Learning, Motivation, and Brain Activity

Since the beginning of the 20th century, researchers have studied how motivation affects learning and vice versa (Mehring and Colson, 1990), since both concepts are reportedly related (Rao, 2003; Crandell and Robinson, 2007). As most students’ scholastic motivation declines with the onset of adolescence (Harter, 1996; Zusho and Pintrich, 2001; Watt, 2004), most students’ learning performance and academic achievement also tend to decrease (Alspaugh, 1998; Barber and Olsen, 2004; Vedder-Weiss and Fortus, 2012). One prominent theory on learning is the reinforcement theory, which is based on the early work of Thorndike (1929) and Skinner (1938). Their theory of operant conditioning posits that individuals learn according to the outcomes of their actions. Specifically, if an outcome – for example good learning performance at school – is reinforced through reward (e.g., praise and feedback from peers and/or teachers), then the corresponding behavior (e.g., good learning performance) is likely to be repeated and vice versa. This means that students’ motivation to learn is directly increased through reward. In turn, punishment can work to suppress certain kinds of undesirable behavior (see Ittel et al., 2013). Both the concept of socio-motivational (in-)dependence and reinforcement theory focus on the interactions between an individual and his or her environment and constitute a promising approach to examine individual differences in motivation *and* learning patterns. Moreover, linking these theories to neuroscience offers the possibility of elucidating individual characteristics beyond mere behavioral aspects. Since learning from reward and punishment represents the fundament also for social learning, investigating the neurobiological correlates of these learning mechanisms may help to explain why some individuals depend on the reinforcing aspects of scholar motivators (peers and teachers), while others do not. Moreover, in neuroscientific experimental settings, the detailed learning experience (i.e., trial-by-trial learning) within the brain can be investigated directly. Here, the combination of functional magnetic resonance imaging (fMRI) and reinforcement learning paradigms such as reversal learning tasks (Cools et al., 2002) constitute a well-established research method to investigate simple operant learning and allow researchers to consider potential inter-individual differences on a neural level during a basic learning task. Reversal learning assesses an individual’s ability to develop advantageous learning behavior by using feedback on performance. Standard 2-choice reversal learning tasks present participants with two potential responses with different reinforcement contingencies. Through multiple trials, participants learn to choose the stimulus associated with a higher reward by referring to their performance feedback. Next, the reinforcement contingency is altered without warning participants, who are thus surprised to discover that their previously reinforced response does not yield a reward anymore, cueing them to switch to the alternative response. As soon as one response is no longer being rewarded, the alternative response always becomes the better choice (see D’Cruz et al., 2011). Using such tasks, brain regions that are crucially involved in signaling learning parameters such as the prediction error (PE) have been identified (O’Doherty et al., 2003; Schlagenhauf et al., 2013). The PE is defined as the difference between an expected outcome and the actual outcome (Sutton and Barto, 1998). These PEs have been found to be encoded in the subcortical striatum (O’Doherty et al., 2003, 2004; D’Ardenne et al., 2008), as well as in prefrontal and parietal areas (O’Doherty et al., 2003; Tobler et al., 2006). The striatum which comprises of putamen and caudatus is known to be involved in basic forms of feedback processing and flexible learning processes (Beeler et al., 2014). The prefrontal cortex (PFC) on the other hand is involved in more controlled *social* feedback learning processes (see Guyer et al., 2012) and higher order control functions (Wood and Grafman, 2003; van Schouwenburg et al., 2010; Wolfensteller and Ruge, 2012).

Students’ motivation to learn is influenced by basic processes such as operant learning. Investigating these processes in experimental neuroscientific studies may help to explain why some individuals depend on reinforcing aspects of scholar motivators and others do not. Understanding these neural processes may constitute the basis for the understanding of aberrant behavior in the school context (e.g., conduct disorders).

### Research Aims and Hypotheses

Here, we used computational modeling to estimate PE values based on the behavioral data of each individual. This approach also provides meaningful parameters that quantify different aspects of learning behavior like the individually estimated learning rate. The learning rate describes how strongly single feedback events influence future choice behavior, i.e., show if an individual adjusts his or her behavior quickly according to the received feedback or if his or her expectations are more stable and only influenced through feedback over a longer period of time. Possibly, since individuals with socio-motivational dependence adapt their motivation and learning to peers and/or teachers (Hoferichter and Raufelder, 2014), an association of individual learning rate values with the probability of being socio-motivationally dependent rather than socio-motivationally independent is suggested.

Furthermore, we hypothesize that PE related activation measured using fMRI during the reinforcement learning task is associated with the individual socio-motivational type. However, since this is the first study to link students’ MT, learning rate and brain activation during a reinforcement learning task, our approach was exploratory.

The main goal of the present study was to explore the interplay of reinforcement learning and socio-motivational (in-)dependence in adolescent students by: (1) investigating whether students’ individual learning rates in a reversal learning task can predict their respective MT, and (2) investigating whether PE related activation in the PFC and the striatum – brain regions that are known to be crucially involved in reinforcement learning – predict the probability of belonging to specific MTs.

## Materials and Methods

### Participants and Procedure

A subsample of 88 mentally and physically healthy (as confirmed by a semi-structured interview assessing psychiatric health care utilization and psychiatric family history) adolescents (*M*_age_ = 15.03; *SD* = 0.51; 44 girls) from 9th grade in secondary schools in the German federal state of Brandenburg was selected from a larger sample of a former quantitative study (*N* = 1088; *M*_age_ = 13.7 years; *SD* = 0.53) to participate in an fMRI study on reinforcement learning by using a reversal-learning task. The 88 participants were chosen according to their high probability (>0.85) of being either (1) a peer-dependent MT (*n* = 20; girls = 12), (2) a teacher-dependent MT (*n* = 17; girls = 10), (3) a peer-and-teacher-dependent MT (*n* = 24; girls = 11), or (4) a peer-and-teacher-independent MT (*n* = 24; girls = 12). This probability was based on ratings on the scales “Peers as positive motivators” (PPMs) and “Teacher as positive motivators” (TPMs), which formed the empirical basis of our preliminary LCA (see Raufelder et al., 2013b). In other words, the sample was highly representative of each MT.

The fMRI sessions were held between June and December 2012. Prior to their fMRI session each student within the current study’s subsample answered questions (among others) about their perception of PPMs and TPMs for a second time, which formed the empirical basis of the typology in the present sample. Since we were particularly interested in students’ own views and perceptions of their socio-motivational relationships with teachers and peers, the questionnaires used for this study were based on self-reports. Three of the 88 participants needed to be excluded: one due to missing data, one due to excessive head movements (more than 3 mm translation or 3° rotation) and one due to neurological abnormalities. The remaining 85 participants were included in the following analysis.

Prior to the fMRI study, participants were thoroughly screened for MRI exclusion criteria (e.g., non-removable ferromagnetic material). Both the participant and one parent or custodian provided their informed, written consent. Participants were free of drug use as well as any medication potentially affecting brain responses. According to the Edinburgh Handedness Inventory (Oldfield, 1971) 81 participants were right handed and four were left-handed. The study was performed in accordance with the latest version of the Declaration of Helsinki, and approved by the ethics committee of the German Psychological Society.

### Questionnaire-Based Measures

#### Teachers as Positive Motivators

This subscale was taken from the Relationship and Motivation (REMO) scale (Raufelder et al., 2013a). TPM consists of six items that showed a reliability of α = 0.81 in the current sample. Students were asked to answer statements such as “I will make more effort in a subject when I think that the teacher believes in me” or “When a teacher helps me, I try to do well in the subject” on a 4-point Likert scale from 1 (*strongly disagree*) to 4 (*strongly agree*).

#### Peers as Positive Motivators

This subscale is also part of the REMO Scale (Raufelder et al., 2013a) and consists of nine items (e.g., “When my friends learn, I am also motivated to learn” or “My friends and I motivate each other to make an effort at school”). Responses were collected using a four-point Likert scale from 1 (*strongly disagree*) to 4 (*strongly agree*) (α = 0.86).

### Neuroimaging Procedure

#### Functional Magnetic Resonance Imaging

Using a 3.0 Tesla Siemens Magnetom Tim Trio scanner with a 12 channel head coil, gradient echo T2^∗^-weighted echo-planar images (EPIs) were acquired while participants performed the reversal learning task (outlined below). 633 EPI volumes containing 38 slices were measured using the following parameters: repetition time (TR) = 2060 ms, echo time (TE) = 30 ms, slice thickness 2.5 mm, matrix size 64^∗^64, field of view (FOV) 224^∗^224 mm^2^, in-plane voxel resolution 3.5 mm^2^, flip angle 80°. In order to correct for disparities in the magnetic field, distortion maps were measured prior to the EPI sequence [TR = 434 ms, TE = 5.19 ms (first) and 7.65 ms (second), slice thickness 3.5 mm, matrix size 64*64 and FOV 224*224 mm^2^, voxel size 3.5^∗^3.5^∗^3.5 mm^3^, flip angle 60°] and included in the analyses. A 3D anatomical image was obtained using a T1-weighted 3D spoiled-gradient echo pulse sequence with TR = 1900 ms, TE = 2.52 ms, matrix size 256^∗^256, FOV 256^∗^256 mm^2^, voxel size 1^∗^1^∗^1 mm^3^, flip angle 9°. Functional images and field-distortion-maps were tilted by 25°.

#### Probabilistic Reversal Learning Task

**FIGURE 1:**
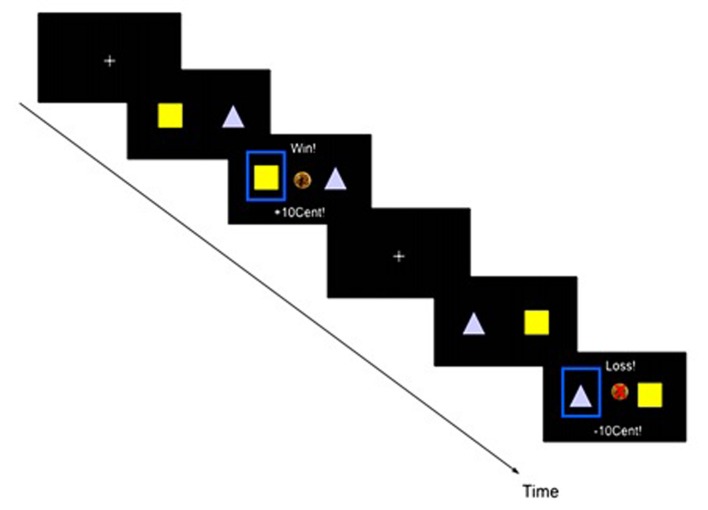
**Probabilistic reversal learning task: one trial consists of stimulus presentation with a response time window (1.5 s), feedback (1 s) and an inter-trial interval of 1.5–6.5 s introducing an exponentially distributed jitter.** Choosing the currently ‘good’ stimulus leads to a reward with a higher probability (80%) than the alternative option (20%). After achieving the criterion of five correct answers out of the last six trials, the chance of a reversal of the probability distribution becomes 20% for the following trials.

During the fMRI, participants performed 300 trials of a probabilistic reversal learning task. In this reinforcement learning paradigm, participants can win money by choosing one of two different stimuli, one of which has a higher probability of yielding a reward. These probabilities reverse during the experiment, therefore the participants have to keep learning and adjusting their choice behavior over the course of the experiment. One of the stimuli was associated with an 80% probability of a monetary reward and a 20% probability of a loss. Inverse probabilities were assigned to the other stimulus. Each trial consisted of three phases: presentation of the two stimuli (maximum 1.5 s), selection of one stimulus by the participant (1.5 s – RT), and feedback (1 s, see **Figure 1**). During the jittered, exponentially distributed inter-trial interval (1–6.5 s) a fixation cross appeared. Then, two symbols were randomly assigned to the left and right-hand side of the screen. Participants had to select one of them by pressing a button within the presentation time window; otherwise the message “Too slow!” (in German “Zu langsam!”) appeared and the experiment proceeded with the next trial. After the button press, a blue frame highlighted the selected target and the feedback (positive or negative) appeared: (1) positive feedback was displayed with an image of a 10 Euro-cent coin accompanied by the message “Win! +10Cent” (in German “Gewonnen! +10Cent”), (2) negative feedback was displayed with a crossed out 10-Euro Cent image accompanied by the text “Loss! –10Cent” (in German “Verloren! –10Cent”). After achieving the criterion of five correct answers (i.e., choosing the stimulus with currently higher chance to win) for the last six trials in a sliding window, the chance of reversal of the probability distribution became 20% for the following trial. Before performing the task in the scanner, students were introduced to the task with the help of a power-point presentation and given a short training session without reversals, in order to be familiarized with the probabilistic nature of the experiment. They were informed about the possibility of changes in reward contingencies during the main experiment and that they would receive the money they won during the task. Participants were instructed to maximize their reward. After performing the task with reversals in the fMRI scanner, participants received their total monetary gain (maximum 8 €).

### Data Analysis

#### Behavioral Data Analysis of Reversal Learning Task

Matlab 2010b (The MathWorks, Natick, MA, USA) and SPSS 19 (SPSS, Inc., Chicago, IL, USA) were used to analyze the behavioral data generated by the reversal learning task. Each participant’s behavioral performance was determined by the proportion of his or her “correct” responses, i.e., choosing the symbol with the currently higher probability to be rewarded.

#### Reinforcement Learning Algorithm

A reinforcement learning model was used to estimate learning parameters that describe individual behavior in the reversal task, and to generate single trial PEs as regressors for the analysis of fMRI data. In detail, a modified Q-learning algorithm was used that calculates five free parameters for each participant that best capture the student’s observed choice behavior. This algorithm updates an expected value (Q-value) based on the outcome of previous trials (Sutton and Barto, 1998). At each trial t, Q-values for the chosen option c were adjusted according to the feedback received:

$$Q_{c,t}=Q_{c,t-1}+{\alpha \delta }_{Qc,t-1}$$

The individual learning rate α determines how quickly expectations change with respect to the current PE δ, which is defined as the difference between the expected and the actual reinforcement R_c,t_:

$$\delta_{Qc,t}=R_{c,t}-Q_{c,t}$$

R_c,t_ indicates two separate free parameters: instead of coding reward and punishment as 1 and −1 respectively, the parameters varied individually for reward and punishment (Schlagenhauf et al., 2014).

In addition, the model estimates the degree to which a participant updates values for the unselected response Q_u,t_. Since the reversal learning task included inversely correlated reward probabilities, double update models are best suited to explain the observed behavior (Glascher et al., 2009; Hauser et al., 2014; Schlagenhauf et al., 2014). Because we were particularly interested in examining inter-individual differences in behavior (i.e., learning), the extent to which a participant utilized a double update strategy was allowed to be weighted by parameter κ:

$$Q_{u,t}=Q_{u,t-1}-{\kappa \alpha \delta }_{Qu,t-1}$$

To optimize the model fit, another free parameter Q_*i*_ was included that specified the initial Q-values for one option (a bias to initially choose one stimulus over another; Schlagenhauf et al., 2014). The probability of choices based on the model-derived values was estimated by means of a softmax. The softmax equation calculates *p*_*a*(*t*)_ – the likelihood of a subject choosing action a over b in trial t – which is assumed to be proportional to the expected value of this option.

$$p_{a}\left(t\right)=\frac{\text{exp}\left(Q_{a}\left(t\right)\right)}{\text{exp}\left(Q_{a}\left(t\right)\right)+\text{exp}\left(Q_{b}\left(t\right)\right)}$$

The set of 5 free parameters was fitted individually for each participant by applying expectation-maximization with empirical priors, and the model evidence was approximated by integrating out the free parameters over the likelihood by sampling from the prior distribution (Huys et al., 2011, 2012). The choice behavior of all but one participant could be explained better than if it had been left up to chance (i.e., based on the likelihood that the observed data are given by the parameters). One participant’s performance was more than three standard deviations below the mean and thus that participant was excluded from further model-based analysis. As such, time-series based on individual parameters can be regressed against imaging data in a meaningful way.

#### fMRI Data Analyses

Functional magnetic resonance imaging image processing and data analyses were performed using Statistical Parametric Mapping software package (SPM8, Wellcome Trust Centre for Neuroimaging, London, UK^1^) in Matlab R2010b (The MathWorks, Natick, MA, USA). Initially, the following image preprocessing steps were conducted: correction for differences in slice time acquisition and motion including unwarping, co-registration of the mean EPI with the anatomical image, spatial normalization, and segmentation into tissue classes of the T1 image using the unified segmentation approach (Ashburner and Friston, 2005). EPIs were spatially smoothed with an isotropic Gaussian kernel of 6 mm full width at half maximum. The general linear model approach used by default in SPM8 was applied. Data analysis was performed following an event-related approach. On the single-subject-level, a regressor was used to model reactions to feedback and the PEs derived from the learning model were added as a parametric modulator. In order to account for movement associated variance, the six rigid body movement parameters and their first temporal derivative as well as a regressor marking scans with more than 1 mm scan-to-scan movement were included in the model as additional regressors of no interest (Iglesias et al., 2013). Individual PE contrast images were taken to a random effects group-level analysis (one-sample *t*-test for within group analysis). All results are reported using family wise error (FWE) correction for the whole brain (*p* < 0.05). For statistical analysis, the blood-oxygen-level dependent (BOLD) parameter estimates (reflecting changes in the concentrations of oxy- and deoxyhemoglobin within the brain and thus indirectly indicating changes in neural activity) were extracted from clusters that were significantly activated [regions of interest (ROI)]. Based on previous studies and to reduce the number of tested areas we focused on the PFC and striatum (Cools et al., 2002; D’Cruz et al., 2011; Schlagenhauf et al., 2013). Extracted values from functional clusters were averaged. Based on studies showing differential activation of striatal subregions during reinforcement learning (O’Doherty et al., 2004), especially in adolescents (Cohen et al., 2010), we additionally extracted parameter estimates from striatal subregions (limbic, sensorimotor and associative striatum; Martinez et al., 2003) to analyze these in more detail.

#### Multinomial Logistic Regression Analysis

The three-step approach of latent class (LC) regression introduced by Vermunt (2010) was used to predict socio-motivational (in-)dependence: in the first step, the LC model is built on students’ replies to PPM and TPM before the fMRI session, although the regression and the LC model are combined into one model. In the second step, similar to LC analysis without covariates, individuals are assigned to the LCs based on their prior MT membership probabilities obtained from step one. The estimated mean allocation probabilities for participants in the current study are above 0.96. However, assigning individuals to single MT categories may generate misclassification errors, since membership probabilities are not always exactly one. In Vermunt’s (2010) three-step approach, the MT categories allocated in step two serve as a single response variable with known measurement error probabilities. Finally, in the third step, a multinomial logistic regression analysis (MLRA) is conducted using the MT category assignment from step two as the observed dependent variables. Thereby, in contrast to MANOVA, the misclassification errors in the LCs are taken into account. The strength and advantages of Vermunt’s three-step approach have been successfully demonstrated (Vermunt, 2010; Asparouhov and Mutheìn, 2012).

The LC model was estimated using three parcels of the original six items (three parcels consisting of two items) of the REMO subscale, TPM, and the original nine items (three parcels consisting of three items) of the REMO subscale PPM, which students filled out immediately before their fMRI session. To ensure that all measurement information enters the structural equations, random parcel building is often used in psychological research (Nasser-Abu and Wisenbaker, 2006). Although some authors argue that parceling is inappropriate in confirmatory factor analysis models (Marsh et al., 2013), others (e.g., Little et al., 2002; Nasser-Abu and Wisenbaker, 2006) underline the advantages of parcel construction due to preventing potential variance sharing and spurious correlations. To predict the extracted LCs, the independent variables deployed in the logistic regression were the learning rate α as well as the extracted values from the fMRI analysis from the ROIs PFC, caudate/thalamus and putamen (left and right hemisphere separately). All analyses were carried out in Mplus 7 (Muthén and Muthén, 1998-2010) with MLR estimator, which is recommended for standardized questionnaire-based analyses with small and medium sized samples (*N* < 100; Wang and Wang, 2012), although the sample size is relatively large for fMRI standards. To account for missing data, the models were estimated using full information maximum likelihood in Mplus.

## Results

### Latent Class Analysis

**Table 1** shows the model fit results for LCA with 2–5 classes. Judging from the Akaike Information Criteria (AIC) and Bayesian Information Criteria (BIC; lowest value), the 4-class solution reveals the best fit for our data. In addition, the Bootstrap Likelihood Ratio Tests (BLRT) indicate that the 5-class solution is not superior to the 4-class solution model (see **Table 1**).

**Table 1:** Model fit results LCA.

	Statistical criteria		
	BIC	AIC	BLRT
Model 1: 2 classes	604.067	573.264	0.000
Model 2: 3 classes	574.430	527.041	0.000
**Model 3: 4 classes**	**561.773**	**497.798**	**0.000**
Model 4: 5 classes	582.656	502.095	1.00

*BIC, Bayesian Information Criteria; AIC, Akaike Information Criteria; BLRT, Bootstrap Likelihood Ratio Test. Statistical fit criteria of the superior model are bold.*

**FIGURE 2:**
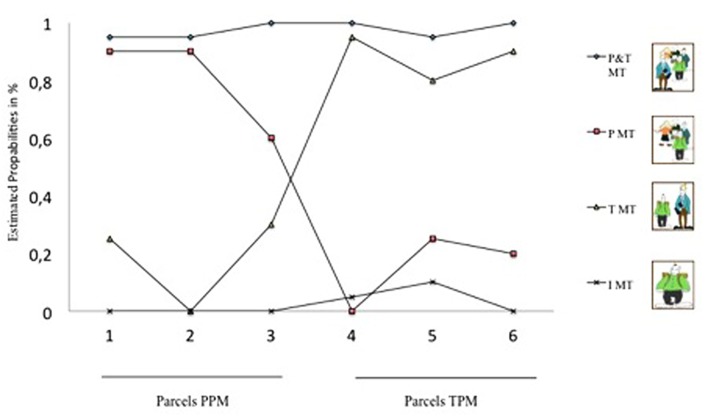
**Latent class analysis of socio-motivational (in-)dependence.** X-axis shows three PPM parcels (1–3) and three TPM parcels (4–6) included in the model analyses. Y-axis shows probability of agreement to the parcels. P&T MT, peer-and-teacher-dependent MT; P MT, peer-dependent MT; T MT, teacher-dependent MT; I MT, peer-and-teacher-independent MT.

The LCA replicated the four MTs (see **Figure 2**): (1) teacher-dependent MT, (2) peer-dependent MT, (3) teacher-and-peer-dependent MT and (4) teacher-and-peer-independent MT. Membership to this 4-class solution was as follows: 22.9% teacher-dependent MT, 26.9% peer-dependent MT, 24.2% peer-and-teacher-dependent MT, and 26.0% peer-and-teacher-independent MT.

### Multinomial Logistic Regression Analysis

**Table 2** shows the results of the MLRA, which tested the learning rate α as well as the extracted values from the PE related BOLD signal of two brain regions that were selected *a priori* based on previous research and due to their involvement in learning processes (PFC and striatum). In addition, to test the association with striatal activation in more detail, the activation of different striatal subregions (associative, limbic, and sensomotoric striatum) during the reversal task was tested as a predictor of the four different MTs. By default, Mplus estimates so-called logit-regression values (transformation form of probability; B-values), since nominal scaled variables (class variables) have no unit. To facilitate interpretation, odds ratios (ORs) have been estimated, which are presented in **Table 2** in second position. Since the peak of ORs is 1, values above 1 increase the probability, whereas values below 1 reduce the probability of membership to one LC (Szumilas, 2010).

**Table 2:** Results multinominal logististic regression.

	Reference class 1			Reference class 2		Reference class 3
	C2	C3	C4	C3	C4	C4
**Learning rate as predictor of four MTs**						
Learning Rate α	−2.98/0.05	−6.99/0.00	−7.71/0.00*	−4.00/0.02	−4.73/0.00	−0.73/0.48
**PE related functional activation as predictors of four MTs**						
Put L	0.05/1.05	0.79/2.20	0.87/2.39	0.73/2.08	0.82/2.27	−0.73/0.48
Put R	0.02/1.02	−1.48/0.23	−0.81/0.44	−1.50/0.22	−0.83/0.44	0.67/1.95
CaTha L	0.99/2.69	1.12/3.06	0.79/2.20	0.27/1.31	−0.20/0.82	−0.34/0.71
CaTha R	−0.73/0.48	−0.21/0.81	−0.22/0.80	0.63/1.88	0.51/1.67	−0.01/0.99
PFC L	−0.62/0.54	−0.17/0.84	0.04/1.04	−1.35/0.26	0.67/1.95	0.21/1.23
PFC R	−0.06/0.94	−1.15/0.32	−1.50/0.22*	0.51/1.67	−1.44/0.24	−0.35/0.70
**PE related functional activation of the different striatum areas as predictors of four MTs**						
As Stri L	4.97/144.03	7.90/2697.28*	6.80/897.85*	2.92/18.54	1.83/6.23	−1.10/0.33
As Stri R	−2.64/0.07	−2.50/0.08	−2.07/0.13	0.14/1.15	0.57/1.77	0.43/1.54
Li Stri L	−1.98/0.14	−1.56/0.21	−3.93/0.02	0.43/1.54	−1.95/0.14	−2.38/0.09
Li Stri R	−0.10/0.90	−1.61/0.20	−0.05/0.95	−1.50/0.22	0.05/1.05	1.56/4.76
Se Stri L	1.09/2.97	−0.67/0.51	−0.78/0.46	−1.77/0.17	−1.88/0.15	−0.11/0.90
Se Srti R	−0.54/0.58	−0.66/0.52	0.69/1.99	−0.12/0.89	1.23/3.42	1.35/3.86

*Class 1 (C1) = Independent MT; Class 2 (C2) = Peer-dependent MT; Class 3 (C3) = Teacher-dependent MT; Class 4 (C4) = Peer-and-Teacher-dependent MT; Put L = Putamen Left, Put R = Putamen Right, CaTha L = Caudate/Thalamus Cluster Left, CaTha R = Caudate/Thalamus Cluster Right, PFC Left = Prefrontal Cortex Left, PFC Right = Prefrontal Cortex Right; As Stri L = Associative Striatum Left; As Stri R = Associative Striatum Right; Li Stri L = Limbic Striatum Left; Li Stri R = Limbic Striatum Right; Se Stri Left = Sensomotoric Striatum Left; Se Stri Right = Sensomotoric Striatum Right; First Position: Logit-Regression values (B), Second Position: Odds Ratios (OR); ^∗^p < 0.05; ^∗∗^p < 0.01; ^∗∗∗^p < 0.001. Bold values are significant.*

### Learning as Predictor of Four Different MTs

The four MT did not differ in their performance measured as percent correct responses, i.e., choosing the symbol with the currently higher probability to be rewarded (*F* = 0.13, *p* = 0.94). The computational model provided individual parameters as a result from the process of fitting the model to each participant’s observed behavior. As depicted in **Table 2**, the individual parameter learning rate α is able to distinguish between the probabilities of being an independent MT rather than a peer-and-teacher-dependent MT (*B* = –7.71, *OR* = 0.00, *p* < 0.05): a high learning rate i.e., a high α value, indicated that a participant tends to be more strongly influenced by the most recent feedback. Such a behavior is associated with membership to the peer-and-teacher-dependent MT rather than the independent MT. Members of the latter category show lower learning rates indicating slower updating of expectations.

### Functional Activation of Learning Regions as Predictors of Four Different MTs

**FIGURE 3:**
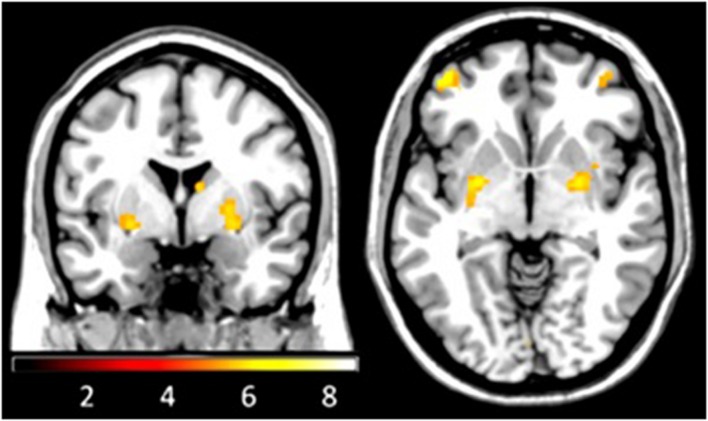
**Functional magnetic resonance imaging during reversal learning.** BOLD signal covaried with prediction errors in a network including the PFC, the parietal cortex, the striatum (putamen and caudate/thalamus-cluster) and the cerebellum (FWE-corrected on the whole brain level, Z-values > 4.8, *p*-values < 0.05).

We found PE related activation in the expected regions, the PFC, the striatum, and in parietal areas, as has been described in previous studies (see **Figure 3**). Activity in the PFC was located in the left inferior and middle frontal gyrus and in the right middle frontal gyrus. The striatum was found to be significantly activated in two separate clusters in each hemisphere, one lateral cluster (putamen) und one dorsal cluster (tail of caudate and anterior nucleus of thalamus).

For the further analysis we used values extracted from regions activated during the reversal learning task in our sample, thereby ensuring that these regions are indeed significantly involved in feedback processing during the task. For an additional analysis focusing on the striatum, a region which receives specific interest regarding PE processing, we used functional masks (Martinez et al., 2003) to extract activation estimates from striatal subregions.

The BOLD signal parameter estimates of two regions (PFC right, and associative striatum left – a subregion of the striatum) that are associated with PE encoding in the reversal task were identified as predictors of the four MTs: our first analysis revealed that PE related activation in the right PFC discriminates between the probability of two categories: the activity level in right PFC predicted the probability of being a peer-and-teacher-dependent MT (*B* = −1.50, *OR* = 0.22, *p* < 0.05) rather than an independent MT. Second, as the results of a separate multinomial regression with the different striatal subareas show, the PE related activation in the left associative striatum discriminates between the probabilities of membership to three categories: the more the activity in the left associative striatum covaried with PEs, the higher the probability of being an independent MT rather than a teacher-dependent MT (*B* = 7.90, *OR* = 2697.84, *p* < 0.05) or peer-and-teacher-dependent MT (*B* = 6.80, *OR* = 897.85, *p* < 0.05). Please see **Table 2** for more details.

## Discussion

The main goal of the current person-oriented, multi-methodological study was to shed light onto individual differences in the interplay between learning and socio-motivational (in-)dependence in adolescence. In particular, reinforcement learning rates as well as activity levels in brain regions that are associated with reinforcement learning (striatum, PFC) were tested as predictors of four different MTs based on the concept of socio-motivational (in-)dependence.

With regard to our first research aim, student’s learning rate indeed functions as a predictor discriminating different MTs, in particular between the peer-and-teacher-dependent MT and the peer-and-teacher-independent MT. In detail, a temporal short term learning rate, characterized by a high α value, is associated with the peer-and-teacher-dependent MT but not with the independent MT. Note that neither a low nor a high learning rate is beneficial in this probabilistic learning task, where an immediate reaction to negative feedback as well as a too slow adjustment of behavior might not increase rewarded events. Thus, students of the peer-and-teacher-dependent MT are more likely to immediately adjust their behavior according to the feedback they receive during the reinforcement learning task. Possibly, this is caused by increased feedback sensitivity in basic learning mechanisms, which could function as a basis for their socio-motivational dependence, i.e., high feedback-sensitivity (recognition and approval) toward peers and/or teachers (Hoferichter and Raufelder, 2014). In turn, the independent MT seems to be less reactive to feedback (reinforcement) during learning as well as in terms of motivation.

However, the learning rate neither discriminated between the three socio-motivational types (peer-dependent MT, teacher-dependent MT, peer-and-teacher-dependent MT), nor between the peer-dependent MT and the teacher-dependent MT compared with the peer-and-teacher-independent MT. Future studies with diverse samples are warranted and may provide deeper insights into these individual differences.

Regarding our second research aim, learning related neural activation in two brain regions (PFC and a subregion of the striatum) discriminated between the probabilities of being socio-motivationally independent as opposed to being socio-motivationally dependent. In detail, more PE related activity in the right PFC predicted the probability of being a peer-and-teacher-dependent MT rather than an independent MT. This finding supports previous research that found the PFC to be associated with more controlled and feedback-related learning processes (Cools et al., 2002; van Schouwenburg et al., 2010; Wolfensteller and Ruge, 2012). In this way, it also supports previous findings on differences in learning-behavior typically found for different MT. In fact, the peer-and-teacher-dependent MT is characterized by a strong feedback-orientation toward both peers and teachers and by well-adjusted and controlled behavior in school (Hoferichter and Raufelder, 2014; Raufelder et al., 2015). Thus, social-motivational dependence might be associated with tendency to follow directions and adapt to feedback in general.

In the additional analysis of subregions within the striatum, we observed that the higher the PE related activity in the left associative striatum, the higher was the probability of being an independent MT rather than a teacher-dependent MT or a peer-and-teacher-dependent MT. During the fMRI experiment, we measure PE related BOLD signals in the striatum. It can be speculated, that individuals from the socio-motivational independent MT group may more strongly rely on their own assumption about their actions and the associated consequences. Such stronger reliance or trust in one’s own estimations of the state of the environment can computationally be understood as higher precision of the PE, which might – perhaps through top down processing – lead to an increase in the measured PE signals in the striatum (Hebart et al., 2016).

Overall, these findings support the concept of socio-motivational (in-)dependence by providing evidence that the concept of socio-motivational (in-)dependence is associated with different learning patterns on (a) a behavioral as well as on (b) a neural level. Following Jensen’s (1998) advice of “teaching with the brain in mind,” our findings revealed that teachers should be aware that student’s individual motivation style [i.e., socio-motivational (in-) dependence] is associated with specific feedback-related brain response during reinforcement learning. Linking these findings to educational practice in order to better support adolescent students and accommodate their individual learning and motivation preferences, students with a socio-motivational dependence benefit from feedback and concrete directions, whereas students with a socio-motivational independence need a more autonomous learning environment with fewer instructions and feedback from teachers and peers. Particularly, students with a socio-motivational independence might not best benefit from the traditional school system, which is dominated through learning in classroom settings and strong teacher involvement and feedback. As strength they show a higher behavioral flexibility, which need to be better supported in class through promoting autonomy-supportive or student-centered teaching behaviors by teachers (Soenens and Vansteenkiste, 2005; Roth et al., 2007; Radel et al., 2010).

### Strengths, Limitations, and Future Directions

Following the person-oriented approach, the present interdisciplinary and multi-methodological study extends existing research on individual differences in the interplay of learning and motivation, specifically the role of reinforcement learning as a predictor of socio-motivational (in-)dependence in adolescence. Compared to current fMRI standards, we studied a relatively large, non-clinical adolescent sample, which provides valid information about neurobiological processes during reinforcement learning considering individual differences in socio-motivational patterns in healthy adolescents. The fMRI design allows giving important insights into adolescents’ brain activity while learning, bridging disciplinary boundaries by combining neuroscientific results to educational psychology in a multi-methodological way. In other words, there are not only individual differences in students’ motivation patterns, but also in their brain activity while learning.

However, some methodological limitations need to be considered when interpreting the current findings. Firstly, the results are limited to German adolescents within the age range of 13–16 years and we are aware that findings may differ for students within another age range, or for students from other countries or different ethnic groups. Secondly, considering the relatively novice field of fMRI-based research on adolescents’ motivational and learning processes, our study is explorative in nature. Future replication studies are warranted to validate our results and to broaden knowledge regarding potential dysfunctions within motivational aspects, learning and its neural basis. Thirdly, one might criticize the use of self-report data. However, studies on motivation that were based on both teacher and student self-reports have reported relative disparity in the information provided by multiple informants (see Skinner and Belmont, 1993). Wentzel et al. (2010) argue that teachers tend to provide invalid information about their students’ perceptions of their own behavior. Since the present study focused on students’ perception of teachers and peers as motivators, a self-report approach is warranted. Moreover, the use of self-report measures has been validated as an appropriate method in psychological research (Chan, 2009). Furthermore, the present study combines self-report data with experimental data, thereby minimizing the weaknesses of each method while maximizing their strengths by combining disparate yet complementary approaches (Raufelder et al., 2012). The reinforcement learning task might not compare directly to the situation in a real classroom setting, however, operant reinforcement learning is one of the most basic forms of learning (see Thorndike, 1929; Skinner, 1938; Ittel et al., 2013) and therefore also constitutes the basis for more complex learning processes in educational settings. The reversal learning paradigm used in the current study is a well-established operationalization of reinforcement learning and is suitable for simultaneous measurement of functional imaging data (Cools et al., 2002). However, we did not examine learning based on social reinforcement but based on more basic secondary reinforcement (money). Based on our findings, future studies are warranted to operationalize this more complex type of (social) reinforcement. Finally, future studies following a person-oriented approach are encouraged to expand existing research on differential motivation and learning patterns (Corpus and Wermington, 2014; Korpershoek et al., 2015) through including neural components. Despite these limitations, this person-oriented study provides deeper insights into the interplay of individual differences in learning and motivational processes in adolescents by using an innovative, multi-methodological, and interdisciplinary research design.

## Author Contributions

DR did the statistical analyses and wrote the main part of the paper. RB did the fMRI analyses of the reversal task and helped writing the paper. LR, SG, RL, TG, and AB were mainly involved in the fMRI procedures including conceptualization, data collection, pre-analyses etc. They additionally helped correcting the manuscript.

## Conflict of Interest Statement

The authors declare that the research was conducted in the absence of any commercial or financial relationships that could be construed as a potential conflict of interest.

## Footnotes

1 http://www.fil.ion.ucl.ac.uk/spm

## References

[B1] Alspaugh J. W. (1998). Achievement loss associated with the transition to middle school and high school. *J. Educ. Res.* 92 20–25. 10.1080/00220679809597572

[B2] Ashburner J. Friston K. J. (2005). Unified segmentation. *Neuroimage* 26 839–851. 10.1016/j.neuroimage.2005.02.018 15955494

[B3] Asparouhov T. Muthén B. O. (2012). *Auxiliary Variables in Mixture Modeling: A3-step Approach using Mplus.* Available at: http://www.statmodel.com/examples/webnote.html

[B4] Barber B. K. Olsen J. A. (2004). Assessing the transitions to middle and high school. *J. Adolesc. Res.* 19 3–30. 10.1177/0743558403258113

[B5] Beeler J. A. Cools R. Luciana M. Ostlund S. B. Petzinger G. (2014). A kinder, gentler dopamine. highlighting dopamine’s role in behavioral flexibility. *Front. Neurosci.* 8:4 10.3389/fnins.2014.00004 PMC3901300 24478624

[B6] Chan D. (2009). “So why ask me? Are self-report data really that bad?,” in *Statistical and Methodological Myths and Urban Legends*, eds Lance C. E. Vandenberg R. J. (New York, NY: Routledge), 309–336.

[B7] Cohen J. R. Asarnow R. F. Sabb F. W. Bilder R. M. Bookheimer S. Y. Knowlton B. J. (2010). A unique adolescent response to reward prediction errors. *Nat. Neurosci.* 13 669–671. 10.1038/nn.2558 20473290 PMC2876211

[B8] Cools R. Clark L. Owen A. M. Robbins T. W. (2002). Defining the neural mechanisms of probabilistic reversal learning using event-related functional magnetic resonance imaging. *J. Neurosci.* 22 4563–4567. 12040063 10.1523/JNEUROSCI.22-11-04563.2002 PMC6758810

[B9] Corpus J. H. Wermington S. V. (2014). Profiles of intrinsic and extrinsic motivations in elementary school: a longitudinal analysis. *J. Exp. Educ.* 82 480–501. 10.1080/00220973.2013.876225

[B10] Crandell J. M. Robinson L. W. (2007). *Low Visions and Blindness.* Springfield, IL: Charles Thomas Publisher.

[B11] D’Ardenne K. McClure S. M. Nystrom L. E. Cohen J. D. (2008). BOLD responses reflecting dopaminergic signals in the human ventral tegmental area. *Science* 319 1264–1267. 10.1126/science.1150605 18309087

[B12] D’Cruz A. M. Ragozzino M. E. Mosconi M. W. Pavuluri M. N. Sweeney J. A. (2011). Human reversal learning under conditions of certain versus uncertain outcomes. *Neuroimage* 56 315–322. 10.1016/j.neuroimage.2011.01.068 21281720 PMC3096874

[B13] Deci E. L. Ryan R. M. (eds) (2002). *Handbook of Self-Determination Research.* Rochester, NY: University of Rochester Press.

[B14] Eccles J. S. Wigfield A. Schiefele U. (1998). “Motivation to succeed,” in *Handbook of Child Psychology. Social, Emotional and Personality Development* Vol. 3 eds Damon W. Eisenberg N. (New York, NY: Wiley), 1017–1096.

[B15] Glascher J. Hampton A. N. O’Doherty J. P. (2009). Determining a role for ventromedial prefrontal cortex in encoding action-based value signals during reward-related decision making. *Cereb. Cortex* 19 483–495. 10.1093/cercor/bhn098 18550593 PMC2626172

[B16] Guyer A. E. Choate V. R. Pine D. S. Nelson E. E. (2012). Neural circuitry underlying affective response to peer feedback in adolescence. *Soc. Cogn. Affect. Neurosci.* 7 81–92. 10.1093/scan/nsr043 21828112 PMC3252630

[B17] Harter S. (1996). “Teacher and classmate influences on scholastic motivation, self-esteem, and level of voice in adolescents,” in *Social Motivation — Understanding Children’s School Adjustment*, eds Juvonen J. Wentzel K. (Cambridge: University Press), 11–42.

[B18] Hauser T. U. Iannaccone R. Ball J. Mathys C. Brandeis D. Walitza S. (2014). Role of the medial prefrontal cortex in impaired decision making in juvenile attention-deficit/hyperactivity disorder. *JAMA Psychiatry* 71 k1165–1173. 10.1001/jamapsychiatry.2014.1093 25142296

[B19] Hebart M. N. Schriever Y. Donner T. H. Haynes J.-D. (2016). The relationship between perceptual decision variables and confidence in the human brain. *Cereb. Cortex* 26 118–130. 10.1093/cercor/bhu181 25112281

[B20] Hoferichter F. Raufelder D. (2014). “Ein Modell inter-individueller unterschiede sozio-motivationaler beziehungen von sekundarschülern mit ihren peers und lehrern,” in *Beziehungen in Schule und Unterricht - Teil 3 Soziale Beziehungen im Kontext von Motivation und Leistung [Relationships in school and class – Part III Social Relationships in context of motivation and achievement]*, eds Tillack C. Fetzer J. Raufelder D. (Immenhausen: Prolog), 170–200.

[B21] Hoferichter F. Raufelder D. Eid M. Bukowski W. M. (2014). Knowledge transfer or social competence? A comparison of German and Canadian adolescent students on their socio-motivational relationships in school. *Sch. Psychol. Int.* 35 627–648. 10.1177/0143034314552345

[B22] Huys Q. J. Cools R. Golzer M. Friedel E. Heinz A. Dolan R. J. (2011). Disentangling the roles of approach, activation and valence in instrumental and pavlovian responding. *PLoS Comput. Biol.* 7:e1002028 10.1371/journal.pcbi.1002028 PMC3080848 21556131

[B23] Huys Q. J. Eshel N. O’Nions E. Sheridan L. Dayan P. Roiser J. P. (2012). Bonsai trees in your head: how the pavlovian system sculpts goal-directed choices by pruning decision trees. *PLoS Comput. Biol.* 8:e1002410 10.1371/journal.pcbi.1002410 PMC3297555 22412360

[B24] Iglesias S. Mathys C. Brodersen K. H. Kasper L. Piccirelli M. den Ouden H. E. (2013). Hierarchical prediction errors in midbrain and basal forebrain during sensory learning. *Neuron* 80 519–530. 10.1016/j.neuron.2013.09.009 24139048

[B25] Ittel A. Raufelder D. Scheithauer H. (2013). “Soziale Lerntheorien,” in *Theorien in der Entwicklungspsychologie [Theories in Developmental Psychology]*, ed. Ahnert L. (Heidelberg: Springer), 330–353.

[B26] Jagenow D. Raufelder D. Eid M. (2015). The development of socio-motivational dependency from early to middle adolescence. *Front. Psychol.* 6:194 10.3389/fpsyg.2015.00194 PMC4340142 25762966

[B27] Jensen E. (1998). *Teaching with the Brain in Mind.* Alexandria, VA: Association for Supervision and Curriculum Development.

[B28] Korpershoek H. Kuyper H. van der Werf G. (2015). Differences in students’ school motivation: a latent class modelling approach. *Soc. Psychol. Educ.* 18 137–163. 10.1007/s11218-014-9274-6

[B29] Little T. D. Cunningham W. A. Shahar G. Widaman K. F. (2002). To parcel or not to parcel: exploring the question, weighing the merits. *Struct. Equat. Model.* 9 151–173. 10.1207/S15328007SEM0902_1

[B30] Marsh H. W. Lüdtke O. Nagengast B. Morin A. J. S. von Davier M. (2013). Why item parcels are (almost) never appropriate: two wrongs do not make a right – Camouflaging misspecification with item parcels in CFA models. *Psychol. Methods* 18 257–284. 10.1037/a0032773 23834417

[B31] Martinez D. Slifstein M. Broft A. Mawlawi O. Hwang D. R. Huang Y. (2003). Imaging human mesolimbic dopamine transmission with positron emission tomography. Part II: amphetamine-induced dopamine release in the functional subdivisions of the striatum. *J. Cereb. Blood Flow Metab.* 23 285–300. 10.1097/00004647-200303000-00004 12621304

[B32] Mehring T. A. Colson S. E. (1990). Motivation and mildly handicapped learners. *Focus Except. Child.* 22 1–15.

[B33] Muthén L. K. Muthén B. O. (1998-2010). *Mplus User’s Guide.* Los Angeles, CA: Muthén & Muthén.

[B34] Nasser-Abu F. Wisenbaker J. (2006). A Monte Carlo study investigating the impact of item parceling strategies on parameter estimates and their standard errors in CFA. *Struct. Equat. Model.* 13 204–228. 10.1207/s15328007sem1302_3

[B35] O’Doherty J. Dayan P. Schultz J. Deichmann R. Friston K. Dolan R. J. (2004). Dissociable roles of ventral and dorsal striatum in instrumental conditioning. *Science* 304 452–454. 10.1126/science.1094285 15087550

[B36] O’Doherty J. P. Dayan P. Friston K. Critchley H. Dolan R. J. (2003). Temporal difference models and reward-related learning in the human brain. *Neuron* 38 329–337. 10.1016/S0896-6273(03)00169-7 12718865

[B37] Oldfield R. C. (1971). The assessment and analysis of handedness: the placeCityEdinburgh inventory. *Neuropsychologia* 9 97–113. 10.1016/0028-3932(71)90067-4 5146491

[B38] Pianta R. C. Hamre B. K. Stuhlman M. W. (2003). “Relationships between teachers and children,” in *Educational Psychology. Comprehensive Handbook of Psychology*, eds Reynolds W. M. Miller G. E. (New York, NY: Wiley), 199–234.

[B39] Radel R. Sarrazin P. Legrain P. Wild T. C. (2010). Social contagion of motivation between teacher and student: analyzing underlying processes. *J. Educ. Psychol.* 102 577–587. 10.1037/a0019051

[B40] Rao M. S. (2003). *Achievement Motivation and Achievement in Mathematics.* New Delhi: Discovery Publishing Hourse.

[B41] Raufelder D. (2014). Pubertät und Lernmotivation [Puberty and learning motivation]. *Lehren und Lernen* 40 16–21.

[B42] Raufelder D. Drury K. Jagenow D. Hoferichter F. Bukowski W. M. (2013a). Development and validation of the Relationship and Motivation (REMO) scales to assess students’ perceptions of peers and teachers as motivators in adolescence. *Learn. Individ. Differ.* 24 182–189. 10.1016/j.lindif.2013.01.001

[B43] Raufelder D. Jagenow D. Drury K. Hoferichter F. (2013b). Social relationships and motivation in secondary school: 4 Different motivation types. *Learn. Individ. Differ.* 24 89–95. 10.1016/j.lindif.2012.12.002

[B44] Raufelder D. Jagenow D. Hoferichter F. Drury K. (2013c). The person-oriented approach in the field of educational psychology. *Prob. Psychol.* 5 79–88.

[B45] Raufelder D. Jagenow D. Hoferichter F. Wilkinson R. P. (2012). An interdisciplinary challenge: method triangulation in the field of brain development and motivation. *Psychol. Res.* 2 627–636.

[B46] Raufelder D. Regner N. Drury K. Eid M. (2015). Does self-determination predict the school engagement of four different motivation types in adolescence? *Educ. Psychol.* 10.1080/01443410.2015.1008405 [Epub ahead of print].

[B47] Roth G. Assor A. Kanat-Maymon Y. Kaplan H. (2007). Autonomous motivation for teaching: how self-determined teaching may lead to self-determined learning. *J. Educ. Psychol.* 99 761–774. 10.1037/0022-0663.99.4.761

[B48] Schlagenhauf F. Huys Q. J. Deserno L. Rapp M. A. Beck A. Heinze H. J. (2014). Striatal dysfunction during reversal learning in unmedicated schizophrenia patients. *Neuroimage* 89 171–180. 10.1016/j.neuroimage.2013.11.034 24291614 PMC3991847

[B49] Schlagenhauf F. Rapp M. A. Huys Q. J. Beck A. Wustenberg T. Deserno L. (2013). Ventral striatal prediction error signaling is associated with dopamine synthesis capacity and fluid intelligence. *Hum. Brain Mapp.* 34 1490–1499. 10.1002/hbm.22000 22344813 PMC3731774

[B50] Skinner B. F. (1938). ‘Superstition’ in the pigeon. *J. Exp. Psychol.* 38 168–172. 10.1037/h0055873 18913665

[B51] Skinner E. A. Belmont M. J. (1993). Motivation in the classroom: reciprocal effects of teacher behavior and student engagement across the school year. *J. Educ. Psychol.* 85 571–581. 10.1037/0022-0663.85.4.571

[B52] Soenens B. Vansteenkiste M. (2005). Antecedents and outcomes of self- determination in three life domains: the role of parents’ and teachers’ autonomy support. *J. Youth Adolesc.* 34 589–604. 10.1007/s10964-005-8948-y

[B53] Sutton R. Barto A. (1998). *Reinforcement Learning: An Introduction.* Cambridge, MA: MIT Press.

[B54] Szumilas M. (2010). Explaining odds ratios. *J. Can. Acad. Child Adolesc. Psychiatry* 19 227–229. 20842279 PMC2938757

[B55] Thorndike E. L. (1929). *Human Learning.* New York, NY: Johnson Reprint Corporation.

[B56] Tobler P. N. O’Doherty J. P. Dolan R. J. Schultz W. (2006). Human neural learning depends on reward prediction errors in the blocking paradigm. *J. Neurophysiol.* 95 301–310. 10.1152/jn.00762.2005 16192329 PMC2637603

[B57] van Schouwenburg M. Aarts E. Cools R. (2010). Dopaminergic modulation of cognitive control: distinct roles for the prefrontal cortex and the basal ganglia. *Curr. Pharm. Des.* 16 2026–2032. 10.2174/138161210791293097 20370667

[B58] Vedder-Weiss D. Fortus D. (2012). Adolescents’ declining motivation to learn science: a follow-up study. *J. Res. Sci. Teach.* 49 1057–1095. 10.1002/tea.21049

[B59] Vermunt J. K. (2010). Latent class modeling with covariates: two improved three- step approaches. *Polit. Anal.* 18 450–469. 10.1093/pan/mpq025

[B60] Wang J. Wang X. (2012). *Structural Equation Modeling: Applications Using Mplus.* Chichester: Wiley.

[B61] Watt H. M. G. (2004). Development of adolescents’ self-perceptions, values, and task perceptions according to gender and domain in 7th- through 11th-grade Australian students. *Child Dev.* 7 1556–1574. 10.1111/j.1467-8624.2004.00757.x 15369531

[B62] Wentzel K. R. (2009a). “Peer relationships and motivation at school,” in *Handbook of Peer Interactions, Relationships, and Groups*, eds Rubin K. Bukowski W. M. Laursen B. (New York, NY: Guilford), 531–547.

[B63] Wentzel K. R. (2009b). “Students’ relationships with teachers as motivational contexts,” in *Handbook of Motivation at School*, eds Wentzel K. R. Wigfield A. (New York, NY: Routledge), 301–322.

[B64] Wentzel K. R. Battle A. Russell S. L. Looney L. B. (2010). Social supports from teachers and peers as predictors of academic and social motivation. *Contemp. Educ. Psychol.* 35 193–202. 10.1016/j.cedpsych.2010.03.002

[B65] Wigfield A. Eccles J. S. (2001). *Development of Achievement Motivation.* San Diego, CA: Academic Press.

[B66] Wolfensteller U. Ruge H. (2012). Frontostriatal mechanisms in instruction-based learning as a hallmark of flexible goal-directed behavior. *Front. Psychol.* 3:192 10.3389/fpsyg.2012.00192 PMC3371695 22701445

[B67] Wood J. N. Grafman J. (2003). Human prefrontal cortex function: Processing and representational perspectives. *Nat. Rev. Neurosci.* 4 139–147. 10.1038/nrn1033 12563285

[B68] Zusho A. Pintrich P. R. (2001). “Motivation in the second decade of life,” in *Adolescence and Education*, eds Urdan T. Pajares F. (Greenwich, CT: Information Age Publishing), 163–200.

